# Optimizing Computed Tomography (CT) for Structurally Complex Organisms: A Case Study of Gastropod Shell Features

**DOI:** 10.1002/ece3.72650

**Published:** 2025-12-19

**Authors:** Andreza Caroline Caiero, Marilia Nagata Ragagnin, Cláudio Campi de Castro, Marcelo Gusmão Paraíso Cavalcanti, Daniel Gorman, Alexander Turra

**Affiliations:** ^1^ Biological Oceanography Department, Oceanographic Institute University of São Paulo São Paulo Brazil; ^2^ Radiology Department, University Hospital University of São Paulo São Paulo Brazil; ^3^ Stomatology Department, School of Dentistry University of São Paulo São Paulo Brazil; ^4^ Commonwealth Scientific and Industrial Research Organization, Environment Canberra Australia

**Keywords:** Artifact, Hermit crab, Imaging technique, Shell, Tomography

## Abstract

The internal volume of gastropod shells is a measured metric in studies of hermit crab ecology, as it determines shell adequacy and subjective value. In this study, we developed and tested an optimized computed tomography (OCT) protocol to estimate shell volume as an alternative to traditional methods, using water or sand. Gastropod species with different shell architectures and sizes were analyzed, and OCT estimates were compared with traditional methods and a previously established nonoptimized CT protocol (NOCT). We also compared the performance of the standard proprietary software (CT Viewer), which operates only on the CT machine computer, with another proprietary program (OsiriX MD), a commercial software. Results showed that OCT produced similar volume estimates to NOCT for high‐spired shells and shells with small elevations on their surface (hips). For low‐spired and middle‐spired shells with less ornamentation, OCT provided higher values than NOCT, and the results were similar to the sand‐filling method. Across species, shell size influenced all methods, with larger high‐spired shells exhibiting greater variability. In contrast, low‐spired shells showed lower variability across all size classes. OsiriX MD yielded estimates equivalent to OCT for the majority of the species and high accuracy and reproducibility. Overall, OCT represents a reliable and nondestructive approach for shell volume estimation, supporting its use in ecological studies. Nevertheless, the NOCT protocol may be more advantageous for species with ornamentation, prickles, or high‐spired shells, as it requires less processing time and does not demand fine adjustments according to species or shell size.

## Graphical abstract

We developed and tested a computed tomography (CT) protocol to estimate shell volume as a method instead of using water and sand. We compared the estimates obtained using this protocol (OCT, optimized CT) with traditional methods (water and sand) and with a CT previous setting (NOCT, nonoptimized CT). We compared the results obtained using proprietary software (attached to the CT machine) with proprietary software (OsiriX MD).

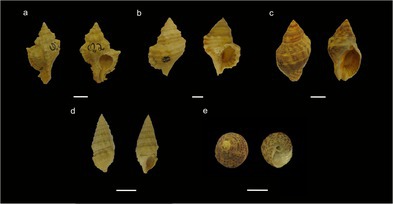

## 1 Introduction

Optimizing scientific methods to provide the most accurate and precise measures of crucial biological parameters is fundamental to comparing studies across time and space (Fritschy et al. 1983; Karlsen and Holm 1994; Angeli et al. 2016; Palanca et al. 2016; Gumsheimer et al. 2017) and to correctly interpret ecological processes (Ragagnin et al. 2018). For this reason, many studies spanning broadly different fields have the goal of validating and improving methods, and this extends to those involving technological and automatized tools, such as imaging techniques (e.g., computed tomography [CT]; Fritschy et al. 1983; Jedenmalm et al. 2011), microcomputed tomography (micro‐CT; Chen, Dall'Ara, et al. 2017; Hambli 2013; Landschoff et al. 2018), scanning electron microscopy (Chen, Holmes, et al. 2017), and ultrasound (Fritschy et al. 1983; Karlsen and Holm 1994; Langeland et al. 2005). Such techniques have been widely used for medical (Kalender 2011), biological (Dhondt et al. 2010; Voss et al. 2011; Flavel et al. 2012; Staedler et al. 2013), and geological studies (Akin and Kovscek 2003; Tracey et al. 2015) because they provide high‐resolution images and precise information in a nondestructive way (Kalender 2011; Rodrigues and Vitral 2007).

Among the different imaging techniques, CT has commonly been used to evaluate structural characteristics (e.g., volume, size, and density) of high‐density structures, such as bones and teeth. CT equipment uses X‐ray measures from the study object to create cross‐sectional images (or thin slices) that are then used to reconstruct a three‐dimensional image of the object using volume reconstruction software (Kalender 2006; Rodrigues and Vitral 2007). Two parameters are then integrated to process the images for analyses: the window level (WL) and window width (WW). The window level is the linear attenuation coefficient and represents the radiation quantity absorbed by the object, distinguishing structures by density (Lontoc‐Roy et al. 2006; Kalender 2011). In contrast, the window width is related to grayscale and contrast of the image, which permits the identification of different body tissues (Johnson et al. 1996; Calhoun et al. 1999). The window level and window width parameters range between 0 and −1024 Hounsfield Units (HU) and from 0 to 4095 HU, respectively. Each element of the built image presents a different value for these parameters; for example, air varies from −100 to −1000 HU, and dense structures such as bones present higher positive values of around 3000 HU (Barnes 1992; Bushberg et al. 2012).

Image reconstruction and analysis is done by the proprietary software coupled with the CT, which may limit the processing time once the equipment is shared among several users. However, images can also be analyzed a posteriori using other image processing software, independently of the CT equipment workstation, using other proprietary or nonproprietary software (Rodrigues and Vitral 2007). The use of proprietary software, such as OsiriX MD, may make the approach cheaper and more accessible, because it is not attached to the CT machine (Bastos et al. 2008), because of its high reproducibility and accuracy, and because it allows an automated method to calculate areas or volumes, decreasing errors (Kim et al. 2012; Shyu et al. 2015). But there is a need to understand the possible convergences or dissimilarities between software and approaches to be applied to specific applications (Gutiérrez et al. 2018).

Image quality is a key element to ensure the precision of measurements and may be improved by adjustment of instrument settings during the analyses (Kalisz et al. 2016). Images are typically processed and analyzed using standard CT settings prescribed by the CT routine, related to tissue type (Souza Junior 1999; Vicente et al. 2004) or according to the image evaluator (Zarelli et al. 2014), which may not be appropriate for studies using objects of different structural types and/or sizes.

CT methods have the potential to be applied in ecological studies (Gutiérrez et al. 2018) and allow the precise estimation of volumes from typically dense structures (Smith et al. 2016). One example of the application of these methods is measuring the internal volume of gastropod shells, which has implications for the behavior of hermit crabs that inhabit them (Ragagnin et al. 2018). These animals depend on empty gastropod shells to protect their vulnerable and noncalcified abdomen (Hazlett 1981; Turra et al. 2005) against biotic and abiotic stressors, such as desiccation (Bertness 1982), osmotic (Conover 1978), and thermal stress (Bertness 1982), and especially predation (Turra 2003; Frameschi et al. 2014). The internal volume is thus extremely important to crab growth and reproductive success or fitness (Briffa and Elwood 2005; Osorno et al. 1998). Shell selection is dependent on the ambient context and the individual size (Bertness 1981; Hazlett 1989; Brown et al. 1993), for example, larger crabs will preferentially select shells with proportionally higher internal volumes to better accommodate in them (Briffa and Elwood 2005). Thus, crabs investigate information on shell internal volume, which has specific ecological significance and should be accurately assessed in studies of ecology and, in particular, behavior.

A range of different methods has been used to measure the internal volume of gastropod shells for studies described in the literature. Most have employed methods that involve filling the shell cavity with sand (Bertness 1981; Hazlett 1989; Brown et al. 1993; Mantelatto and Garcia 2000; Fantucci et al. 2008; Toratti and Mantelatto 2008; Ayres‐Peres et al. 2012) or water (Conover 1978; Osorno et al. 1998; Floeter et al. 2001; Briffa and Elwood 2005; Frameschi et al. 2013, 2014). However, these materials exhibit certain characteristics that mean they may not completely fill the shell cavity, which can often generate substantial errors in estimates (Ragagnin et al. 2018).

CT has been suggested as an alternative approach under the assumption that sand and water methods might underestimate shell volume. However, controlled tests showed that measures derived using this method can be inherent artifacts of the technique (Ragagnin et al. 2018). One of these was associated with the standard CT proprietary software used in image acquisition, processing, and analysis, which may misinterpret the distinction between internal air and the shell wall (Ragagnin et al. 2018).

In order to improve confidence in the results obtained using CT when applied to calculating shell internal volume, it is crucial to optimize the WW and WL settings in a way that will maximize image contrast to improve estimates for different shell species, architectures, and sizes. This procedure may lead to the establishment of a standardized and comparable approach that is independent of the shell species or size, improving the cost‐effectiveness of the method. In this context, we aimed to: (1) establish an optimized protocol for the CT method (OCT) by manipulating window width (WW) and window level (WL) parameters to find the most suitable combination for each shell species, considering architecture and size; (2) test the use of a commercial proprietary software (OsiriX MD) with a standard proprietary software (CT Viewer) as an alternative to the software coupled to the CT workstation; and (3) compare the volume estimates from OCT with sand and water methods and a previous CT setting (NOCT, nonoptimized CT).

## 2 Methods

### 2.1 Biological Models

**FIGURE 1:**
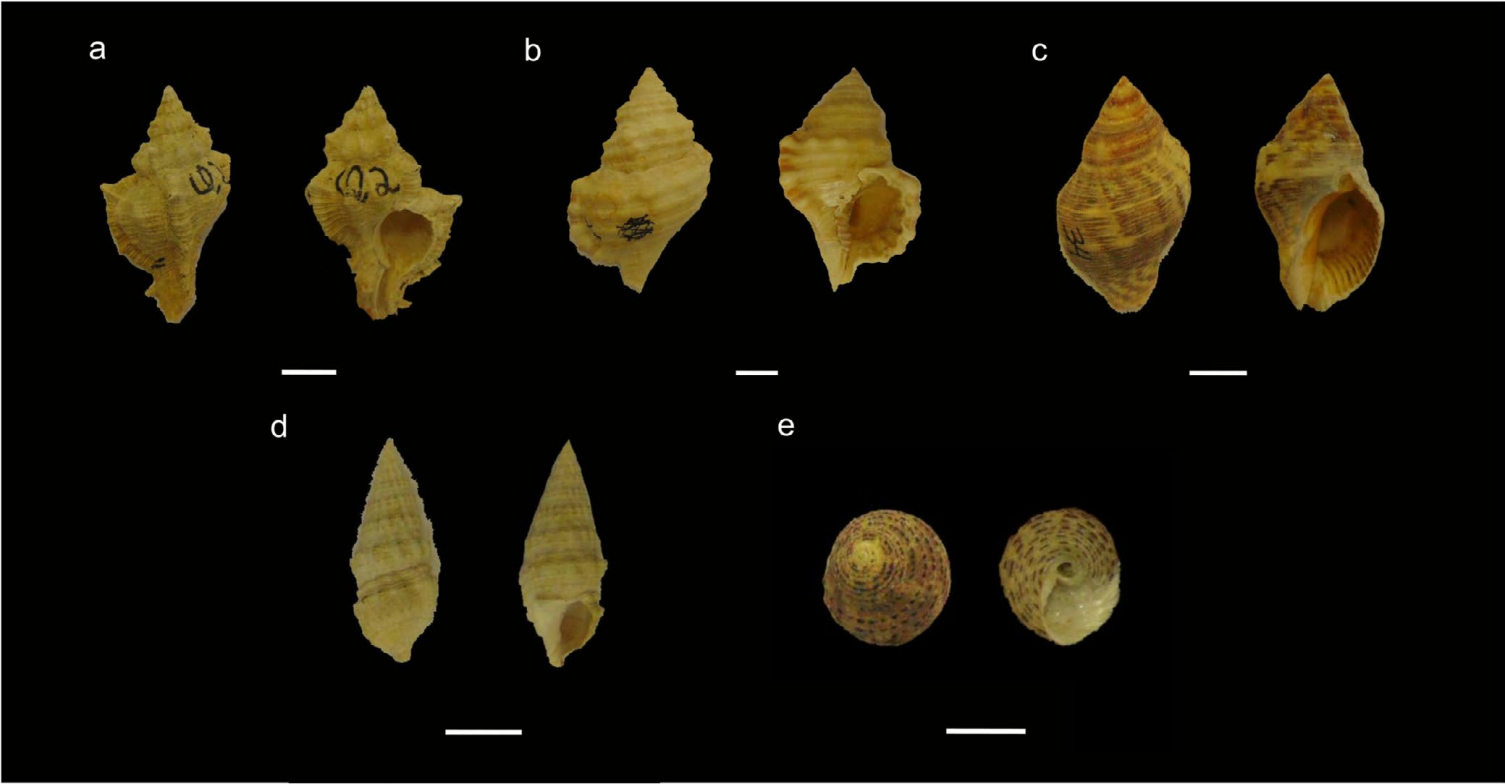
Gastropods shell species used as biological models to optimize computed tomography application to analyze shell internal volume: (a) *Siratus senegalensis (medium‐spired architecture)*; (b) *Monoplex parthenopeu (medium‐spired architecture)*; (c) *Stramonita haemastoma* (medium‐spired architecture); (d) *Cerithium atratum* (high‐spired architecture); and (e) *Tegula viridula* (low‐spired architecture). Scale bar: 10 mm.

Five gastropod species with different shell architectures were used as biological models (Figure 1). The medium‐spired species: *Siratus senegalensis* (Gmelin, 1790), presents prominent princles (Figure 1a); *Monoplex parthenopeus* (Salis Marschlins, 1793), characterized by small elevations, such as hips (Figure 1b); and *Stramonita haemastoma* (Linnaeus, 1767; Figure 1c). The high‐spired species *Cerithium atratum* (Born, 1778), also exhibits small hips on the shell surface (Figure 1d). The low‐spired species *Tegula viridula* (Gmelin, 1791) has a smooth, round shell (Figure 1e). All species occur along the Brazilian coastline (Rios 2009) and are occupied by several hermit crab species (Leite et al. 1998; Mantelatto and Garcia 1999; Turra and Leite 2002; Turra 2003; Sant'Anna et al. 2006; Pereira et al. 2009). Their wide distribution along the Brazilian coast makes them suitable models for the present study.

### 2.2 Establishment of a CT Protocol

#### 2.2.1 Acquisition of the CT Images

To define the optimized protocol, nine gastropod shells were used: three large‐sized medium‐spire shells ( *S. senegalensis* : shell length (SL) = 50.2 mm; *M. parthenopeus*: SL = 50 mm; and *S. haemastoma* : SL = 40.2 mm) and six shells of two species classified in three different size classes: the high‐spired species *C. atratum* (large: SL = 31.7 mm; medium: SL = 20.9 mm; and small: SL = 10 mm) and the low‐spired species *Tegula viridula* (large: SL = 12 mm; medium: SL = 11.3 mm; and small: SL = 3.6 mm).

**FIGURE 2:**
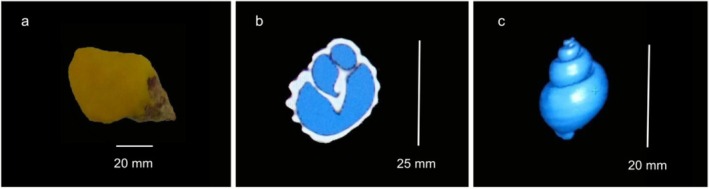
Sequential process to estimate the shell internal volume. (a) the shell of the *Stramonita haemastoma* with the aperture covered by a thin layer of clay; (b) identification of shell internal air (blue) from a sagittal slice of *Monoplex parthenopeus* (parameters combination: WW = 950; WL = −550); and (c) the 3D reconstruction of internal volume of air from *M. parthenopeus*, which represents the shell internal volume.

The CT images of these shells were obtained from a previous study based on a multislice tomography technique using the Philips Brilliance CT 64‐channel scanner (Philips Medical Systems, Amsterdam, The Netherlands; Ragagnin et al. 2018). The settings used in this study to capture the CT images were: 100 mA/slice, 120 kV, 0.640 × 0.625 of collimation, 0.891 of pitch, 0.5 s of rotation time, 54 mm of field of view (FOV), standard filter, standard resolution, 0 of enhancement, 0.67 mm of slices thickness with 0.33 mm of increment and 512 × 512 matrix. The shell aperture was covered by a thin layer of clay to isolate the shell cavity from the external air (Figure 2a).

The images from each specimen were manipulated using the same tomography workstation attached to the tomography equipment and software (CT Viewer). The measurement of volume by CT software is totally automated using tools to select the internal air (i.e., the Region of Interest: ROI) in a cross‐sectional image (Figure 2b), and the internal volume was estimated by the volume of air inside the shell cavity (Figure 2c).

#### 2.2.2 Three‐Dimensional Reconstruction

Two conditions were established for the identification of inadequate image reconstruction for analyses, considering the combination of WW and WL: (1) The presence of external air loss, which also indicates the possibility of internal air loss; and (2) The misinterpretation of the columella (the central structure of gastropod shells that serves as an anchor for the hermit crabs onto the shell) when it is detected as air (and because of that as the estimates of the internal air). The second condition is important to avoid the columella being computed as air because it is not occupied by hermit crabs.

**FIGURE 3:**
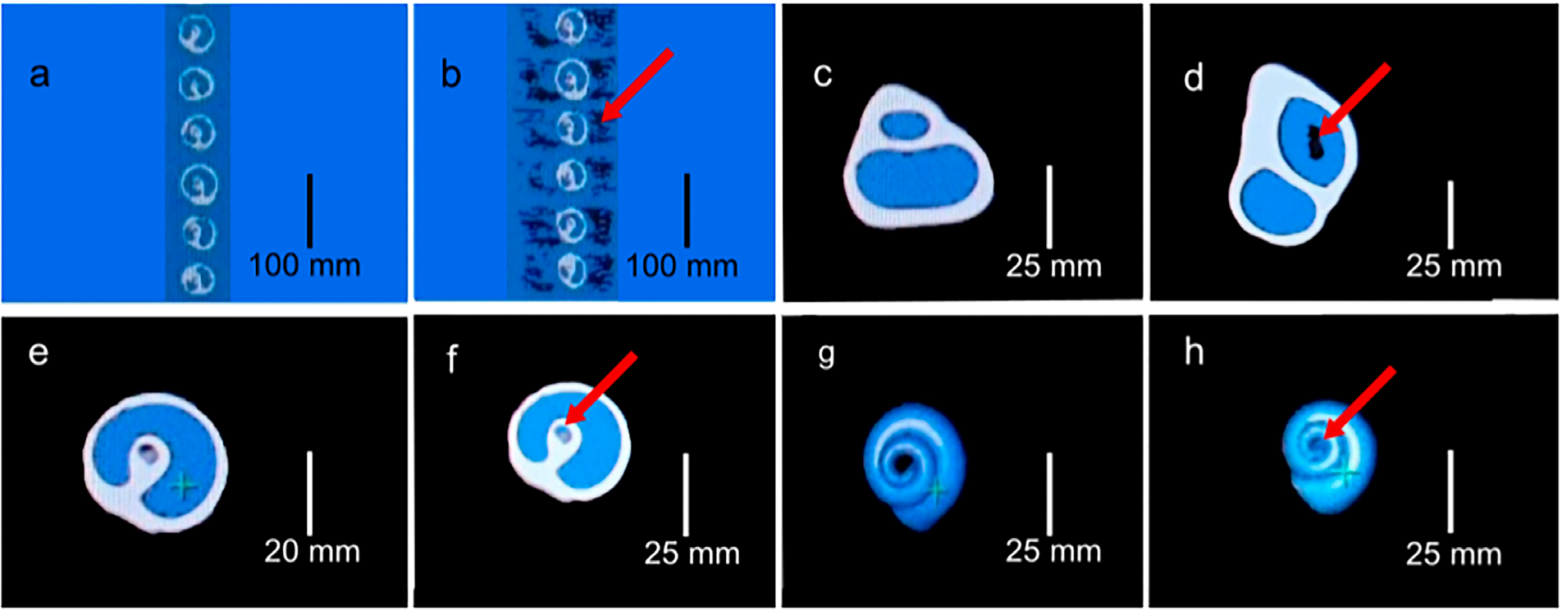
Shell image types used to estimate the internal volume of gastropod shells. Shell structure is indicated in white, and air is indicated in blue. (a) axial slice for a sample of shells used in the study, indicating no loss of external air (blue area); (b) sagittal slice of a sample of the same shells, indicating loss of external air represented by black spots (red arrow); (c) sagittal slice of *Tegula viridula* with no loss of internal air; (d) sagittal slice of *Stramonita haemastoma* indicating loss of internal air (red arrow); (e) axial slice of *T. viridula* when the columella was not considered in shell internal volume calculation; (f) axial slice of *T. viridula* when the columella was considered in the shell internal volume calculation (red arrow); (g) 3D reconstruction of internal air volume of *T. viridula* when the columella was not considered in the shell internal volume calculation; and (h) 3D reconstruction of internal air volume when the columella was considered in the shell internal volume calculation (red arrow).

**FIGURE 4:**
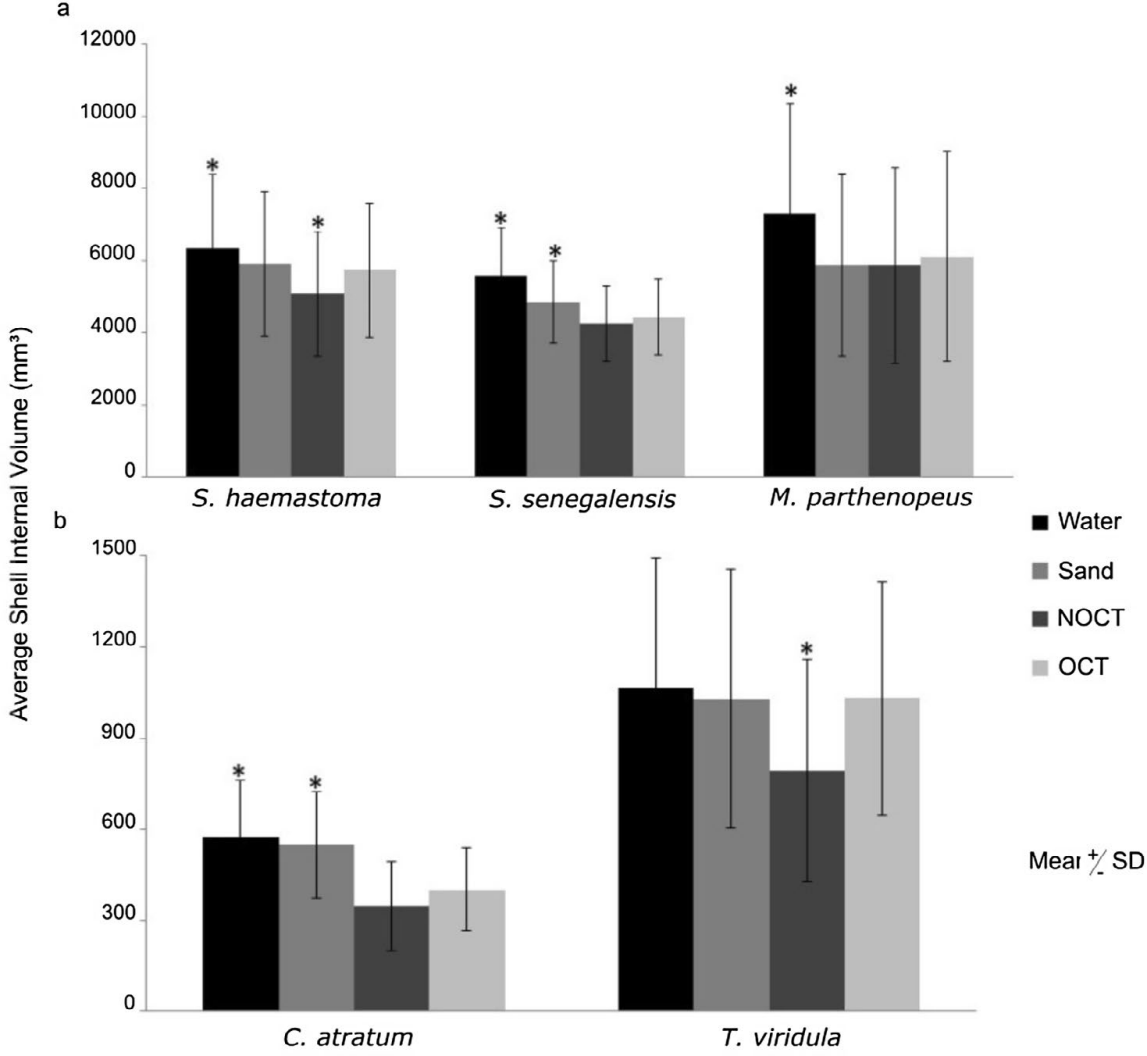
Comparison of the average shell internal volume (mean ± SD) estimated for each method (water, sand, nonoptimized computed tomography (NOCT); and optimized computed tomography (OCT)) for (a) the large shell species: *Stramonita haemastoma*, *Siratus senegalensis*, and *Monoplex parthenopeus* (*n* = 30 each); and for (b) the species exhibiting contrasting shell architectures with size variation, *Cerithium atratum* and *Tegula viridula* (*n* = 20 each). Statistical differences are indicated by the symbol *.

Following these conditions, the images were analyzed from four perspectives in Multi‐planar Reconstruction Images (MPR): (1) axial slices for a sample of shells to verify if the external air was not considered in the reconstruction of the shell cavity (condition hereafter indicated as “loss of external air”; Figure 3a,b); (2) sagittal slices of each shell to verify if there was a loss of internal air *per* specimen (Figure 3c,d); (3) axial slices of each shell to verify if the columella was considered in volume estimation (Figure 3e,f); (4) the 3D reconstruction of the internal volume to ensure that the columella was not considered in estimated values (since in some cases it was not evident in the axial slice, but appeared in the 3D reconstruction; Figure 3g,h).

#### 2.2.3 Manipulating the CT Images

To optimize volume measurements obtained from the CT method according to shell architecture and size (OCT protocol), the settings of WW and WL were changed to evaluate the variation of the estimated volumes. First, WW was fixed, and WL was set every 50 units from 0 to −1024 HU. This procedure was performed every 100 units of WW from 900 to 1800 HU. All these parameter combinations were obtained to detect the settings that would provide the highest volume measurement, following the two conditions above. Preliminary tests indicated that excessive external air loss occurs with WW values lower than 900 HU, while a change in shell thickness occurs with values higher than 1800 HU. Thus, the range of WW was defined according to these results. The range that does not manifest external air loss was defined as “nonexternal air loss range.”

Maintaining a steady WL value, the WW parameter was then ranged every 100 units from 0 to 4095 HU. Then, the WW was ranged every 50 units within this WW range to yield the highest volume value, following the two conditions described above.

#### 2.2.4 The Ideal Parameter Combination

For each tested shell specimen (*n* = 9), these steps were repeated to obtain the highest internal volume and define the ideal parameter combinations. The combination of both established parameters was considered the most appropriate to give the highest accuracy volume estimate for each species and size class. However, this should be regarded as a preliminary ideal parameter combination since a specific set of parameters had to be determined individually for each of the nine specimens.

### 2.3 Application of the OCT Protocol in the CT Method Data

To validate the established protocol for an OCT method, the ideal parameter combinations obtained (Table 3) were applied to a set of specimens (*n* = 163), using the same software (CT Viewer) and workstation as the protocol, as well as the same shell specimens and CT images used in Ragagnin et al. (2018).

We used large specimens of *S. senegalensis* (*n* = 30, SL = 57.5 ± 0.50 mm), *M. parthenopeus* (*n* = 30, SL = 50.9 ± 0.62 mm), and *S. haemastoma* (*n* = 30, SL = 49.2 ± 0.46 mm) and a wide range of shell sizes of *C. atratum* (*n* = 35) and *T. viridula* (*n* = 38) classified as large ( *C. atratum* : *n* = 9, size range: SL = 29.0–34.4 mm; *T. viridula* : *n* = 20, SL = 12.0–19.1 mm), medium ( *C. atratum* : *n* = 12, SL = 20.9–28.9 mm; *T. viridula* : *n* = 9, SL = 9.0–11.8 mm), and small specimens ( *C. atratum* : *n* = 14, SL = 8.5–17.5 mm; *T. viridula* : *n* = 9, SL = 3.3–8.0 mm).

### 2.4 Comparison of Volume Estimates Between Methods

Previous volume estimates derived using sand and water methods (i.e., filling the shell cavity with these materials) and the nonoptimized CT (NOCT) method (i.e., employing the standard parameter combination across all architectures and sizes, WW = 1000 HU and WL = −650 HU) were used in this analysis (data from Ragagnin et al. 2018). Data from these three methods were compared with the estimates obtained using the OCT method. We also included estimates derived from the proprietary software OsiriX MD (Pixmeo, Geneva, Switzerland), tested as an alternative to CT Viewer software (the standard proprietary software coupled to the CT workstation). Statistical analysis involved repeated measures Analysis of Variance (RM‐ANOVA), followed by Tukey test to investigate *post hoc* differences in estimated shell volumes between these four approaches, using the large shell species *C. senegalensis* (*n* = 30), *C. parthenopeum* (*n* = 30), and *S. haemastoma* (*n* = 30). Before analyzing the volumes by statistical methods, the raw data were verified to ensure that the assumptions of the ANOVA were contemplated, normality and equal variance (Levene's test); furthermore, the shells used in the study were randomly selected (ensuring the independence of the data), and the outliers (great volume obtained for little shells or small volume obtained for bigger shells) were removed from the study to avoid analysis distortion. Linear regression analysis comparing the average volume from the water, sand, OCT, and NOCT methods was used to complement the analysis.

Repeated measures Analysis of Covariance (RM‐ANCOVA) was also used to test the effect of shell size (measured as dry weight) and architecture for estimated volumes among the four approaches and the OsiriX software, using specimens across a range of sizes available in nature and characterized by contrasting architectures: *C. atratum* (*n* = 20) and *T. viridula* (*n* = 20). Dry weight was used in preference to shell length because this metric is not comparable among shells of markedly different architecture (low, high, or intermediate spired patterns). Dry weight data were obtained from a previous study where measurement methods are detailed (Ragagnin et al. 2018). The analysis was complemented by linear regression between the average volume from the OCT method and the average volume derived from the water, sand, and NOCT methods.

### 2.5 Estimation of Shell Volume Using OsiriX MD

We used OsiriX MD software as an additional approach to measure shell volume to test whether it could be used as an alternative to the standard CT Viewer that is a computer software attached to the CT scan. This alternative aimed to increase access to the CT method by providing estimates for analogous studies and has the advantage of being a platform that is used in computers not attached to the CT scan (Rosset et al. 2004). The software has a high accuracy and reproducibility ratios (Kim et al. 2012; Shyu et al. 2015), it permits analysis of Digital Imaging and Communications in Medicine Images (DICOM) and has been used to evaluate quantitative measurements in medical (Yamauchi et al. 2010; Fortin and Battié 2012; Kim et al. 2012; Shyu et al. 2015; Gumsheimer et al. 2017) and dentistry studies (Pinheiro et al. 2015; Kobayashi‐Velasco et al. 2017).

The ideal parameter combinations for each species (Table 3) were applied to the same 163 shell specimens through an independent workstation that was not attached to the CT scan (iMac, Apple, Cupertino, California) and located in the 3D Image Laboratory at the School of Dentistry, University of Sao Paulo, Brazil. This workstation had an image resolution of 2560 × 1440 pixels, with images displayed using the DICOM viewer software, OsiriX MD 9.0 64‐bit.

Within the OsiriX software environment, the images can be visualized and manipulated using their axial, sagittal, or coronal slices in the MPR. To select the internal air and estimate volume, we used the Region of Interest (ROI) tool. To select the ROI and replicate it for all slices, the tool “Growing Area” was used with the following parameter settings selected: (1) 3D growing region (entire series); (2) Algorithm: threshold (interval); (3) Interval 1000; (4) Preview result when clicking; (5) Merge with existing Brush ROIs; (6) Propagate result in 4D; (7) Create ROIs in the original series; (8) Brush ROI of points high; and (9) Set outside pixels to 0. The software determines the ROI by selecting a point within the area that corresponds to the air. At this step, the interval must range between negative values (from −100 to −1000 HU) to select only the air. Upon completion of the above steps, the ROI for all shell slices was compiled, and the volume was automatically calculated using the “Measurement Volume” tool in the ROI toolbar.

Two volume measurements were obtained for each shell specimen by the same operator within a 2‐week interval, following the same protocol used in several studies using accuracy and reliability of OsiriX MD (Yamauchi et al. 2010; Kim et al. 2012; Pinheiro et al. 2015; Shyu et al. 2015; Kobayashi‐Velasco et al. 2017) using OsiriX MD. To test if the two contrasting volume measurements from both software (CT Viewer and OsiriX MD) were equivalent, the Intraclass Correlation Coefficient (ICC) was applied (as described in Kim et al. 2012; Gumsheimer et al. 2017; Atli et al. 2018). The analysis was complemented by Pearson Correlation analysis (comparing the average volume from OCT and the average volume derived by OsiriX MD).

## 3 Results

### 3.1 Establishment of the OCT Protocol

The combination of the two established parameters (WW and WL) proved to be the most effective to provide highly accurate volume estimations for each species and size class (Table 1). Although this was regarded as a preliminary ideal, further evaluation was required using the entire set of specimens (*n* = 163 shells).

**TABLE 1:** Preliminary ideal parameter combinations established in Hounsfield Units (HU) for species and size class (*n* = 9 shells).

Shell species and size classes	*N*	WW (HU)	WL (HU)
*S. senegalensis*	1	1000	−650
*M. parthenopeus*	1	1150	−550
*S. haemastoma*	1	2150	−550
*C. atratum*	3		
Large	1	1000	−750
Medium	1	1550	−550
Small	1	1000	−800
*T. viridula*	3		
Large	1	2000	−550
Medium	1	2350	−550
Small	1	1050	−550

*Note:* The protocol was developed for each gastropod shell species (*Sirenatus senegalensis, Monoplex parthenopeus, Stramonita haemastoma, Cerithium atratum*, and *Tegula viridula* ) and size classes (large, medium, and small; only for *C. atratum* and *T. viridula* ).

Abbreviations: *N*, the number of analyzed shells for each species and size class; WL, window level; WW, window width.

### 3.2 Application of the OCT Protocol in the CT Method Data

Despite the established protocol demonstrating the ideal parameters for the 9 shells used as models (Table 1), its application to the other specimens (*n* = 163) revealed that most of the shells required slight adjustment of the settings because, in some cases, there was a disrespect of the two conditions established above. For this reason, a smaller range of ideal parameters was established and applied to each specimen in order to redefine the ideal protocol (Table 2). In this way, the WW and WL that were defined for the final ideal protocol comprised those values that were most widely applicable to the majority of shells (of each specimen) and yielded the most accurate volume estimate (i.e., the highest volume estimate without image artifacts; Table 3).

**TABLE 2:** All parameter combinations ranging in Hounsfield Units (HU) applied throughout the study (*n* = 163 shells), together with the percentage of shell specimens that conformed to each respective parameter‐combination range.

Shell species and size classes	*N*	% of shell specimens	WW (HU)	WL (HU)
*S. senegalensis*	30	100	1000	−650 to −850
*M. parthenopeus*	30	100	1000–1550	−550 to −800
*S. haemastoma*	30	100	1000–2150	−550 to −700
*C. atratum*	35	100		
Large	9	26	1000	−550 to −750
Medium	12	34	1000–1550	−550 to −800
Small	14	40	1000–1250	−550 to −800
*T. viridula*	38	100		
Large	20	52	1000–2000	−550 to −700
Medium	9	24	1000–2500	−550 to −600
Small	9	24	1000–1050	−550 to −600

*Note:* The protocol was established for each gastropod shell species (*Siratus senegalensis, Monoplex parthenopeus, Stramonita haemastoma*, *Cerithium atratum* , and *Tegula viridula* ) and size classes (large, medium, and small; only for *C. atratum* and *T. viridula* ).

Abbreviations: WL, window level; WW, window width.

**TABLE 3:** Final established protocol with the ideal parameter combinations in Hounsfield Units (HU) used in the study (*n* = 163 shells), indicating the percentage of all shell specimens that fit each parameter combination indicated (WW and WL), along with the percentage of specimens from each shell species that matched each parameter individually (WW or WL).

Shell species and size classes	*N*	% of shell specimens	WW (HU)	WL (HU)
*S. senegalensis*	30	93	1000 (100%)	−650 (93%)
*M. parthenopeus*	30	33	1050 (43%)	−750 (40%)
*S. haemastoma*	30	23	1000 (23%)	−550 (63%)
*C. atratum*	35	100		
Large	9	78	1000 (100%)	−750 (78%)
Medium	12	33	1000 (75%)	−750 (33%)
Small	12	33	1000 (75%)	−750 (33%)
*T. viridula*	38	100		
Large	20	40	1850 (60%)	−550 (65%)
Medium	9	56	1000 (78%)	−550 (78%)
Small	9	78	1050 (78%)	−550 (89%)

*Note:* The protocol was established for each gastropod shell species (*Siratus senegalensis, Monoplex parthenopeus, Stramonita haemastoma*, *Cerithium atratum* , and *Tegula viridula* ) and size classes (large, medium, and small; only for *C. atratum* and *T. viridula* ).

Abbreviations: WL, window level; WW, window width.

### 3.3 Comparison of Volume Estimates Using the Different Applied Methods

For the three large shell species with medium spires, volume estimates varied between methods (repeated measures ANOVA; *F* = 343.07; df = 4; *p* < 0.001). Shell species also influenced volume estimates (repeated measures ANOVA; *F* = 3.92; df = 2; *p* = 0.020). A significant interaction was apparent between method and species for these shells (repeated measures ANOVA; *F* = 12.58; df = 8; *p* < 0.001).

The water method demonstrated the highest average volume for: *S. senegalensis* (Tukey test; Water > Sand > NOCT = OCT), 20% and 24% higher than estimates from OCT and NOCT, respectively; *S. haemastoma* (Tukey test; Water > Sand = OCT > NOCT), 7% higher than estimates from sand, 10% than OCT method, and 20% than NOCT; *M. parthenopeus* (Tukey test; W > S = NOCT = OCT), 20% higher than sand method, 16% than OCT method, and 20% than NOCT (Figure 4a). The second highest mean volume estimates were provided by the sand method, which was 8% and 12% higher than OCT and NOCT, respectively for *S. senegalensis* , and 14% higher than NOCT for *S. haemastoma* (Figure 4a).

The average volume estimated through OCT was higher than NOCT (greater by 12%) only for *S. haemastoma*. Further, the volume obtained using OCT was similar to that estimated using sand for this shell species (Figure 4a). Nonetheless, the average volume obtained through OCT was similar to the NOCT method for the most ornamented shell, *S. senegalensis* (Figure 4a). Meanwhile, the average volume from NOCT and OCT presented the same estimates from the sand method for the ornamented shell, *M. parthenopeus* (Figure 4a), suggesting the wide applicability of this method to obtain estimates of the volume of gastropod shells.

Regarding the comparison of species having contrasting shell architecture (low and high‐spired) of varied sizes, the estimated volumes differed among the methods for the species that have size variation (ANCOVA; *F* = 3.0023; df = 4; *p* < 0.05). Species influenced volume estimates for shells exhibiting size variation (ANCOVA; *F* = 14.1135; df = 1; *p* < 0.001), and the significant interaction between method and species (ANCOVA; *F* = 8.32; df = 4; *p* < 0.001) suggested the influence of architecture and method on estimated volumes.

The water and sand methods also demonstrated the highest averages of volume for both species, but with no difference between them for *C. atratum* (Tukey test; Water = Sand > NOCT = OCT). The average volumes obtained by the water and sand methods were 30% and 27% higher than OCT, respectively. The water, sand, and OCT methods demonstrated similar averages for *T. viridula* (Tukey test; Water = Sand = OCT > NOCT), with estimates from water 26% higher than NOCT. Both volumes obtained by sand and OCT were 23% higher than NOCT (Figure 4b). The average volume estimated using OCT was higher than NOCT (23%) only for *T. viridula* and moreover did not differ from the water and sand methods (Figure 4b). For *C. atratum* , the volume estimates from NOCT and OCT were similar (Figure 4b).

**FIGURE 5:**
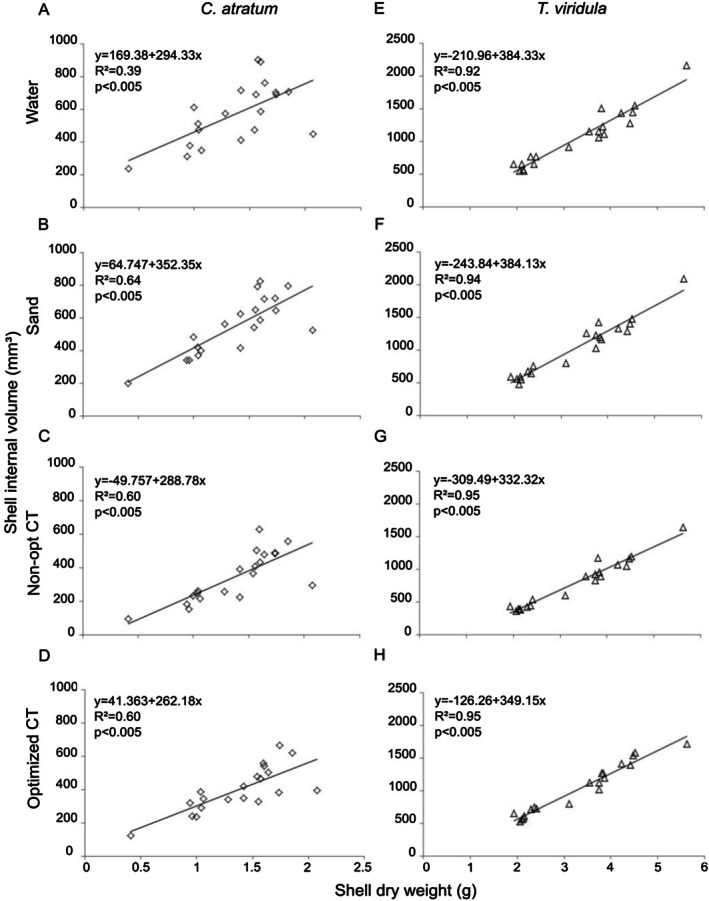
Relationships between shell dry weight in grams (g) and shell internal volume (mm^3^) for the methods: water, sand, nonoptimized computed tomography (NOCT), and optimized computed tomography (OCT) for species exhibiting size variation: (A) water, (B) sand, (C) NOCT and (D) OCT for *Cerithium atratum* ; and (E) water, (F) sand, (G) NOCT and (H) OCT for *Tegula viridula* .

Shell size influenced the volume estimates (ANCOVA; *F* = 384.24; df = 1; *p* < 0.001), with a significant relationship between measured volume and shell dry weight for both *C. atratum* and *T. viridula* across all methods (Figure 5). Large shells of *C. atratum* showed high variability for all methods (Figure 5A–D). Estimates derived using the sand method demonstrated a strong relationship between volume measurements and shell dry weight for *C. atratum* (Figure 5B). NOCT and OCT methods had the same coefficient of determination (*r* ^2^, Figure 5C,D) and were comparable to that of sand. *T. viridula* exhibited less variability for all size classes and methods (Figure 5E–H). All methods had stronger relationships (> 92%) and lower variability. NOCT and OCT methods showed the highest relationship between estimated values and shell dry weight (Figure 5G,H).

The average volume estimates derived from the three methods (water, sand, and NOCT) showed strong relationships (*r* ^2^ > 0.95) with the average volume from OCT for the three large shell species with medium spires (Figure 5A–C), indicating proportional values even with an error or variation. For species for which shell size variation was considered, the most ornamented shell ( *C. atratum* ) showed lower *r* ^2^ values for both methods compared with the other species. The wide spread of points indicates a weaker relationship between the estimates from OCT, especially with those from the water and sand methods (*r* ^2^ = 0.41 and 0.59, respectively), but a comparatively better relationship with NOCT (*r* ^2^ = 0.71; Figure 5D). Moreover, all methods showed strong relationships with OCT when comparing the estimates for the less‐spired shell, *T. viridula* (*r* ^2^ > 0.90 for the three methods), which indicated that the three methods provided proportional estimates (Figure 5E), since the coefficient of relationship is high.

### 3.4 Estimation of Shell Volume Using Commercial Proprietary Software (OsiriX MD)

**FIGURE 6:**
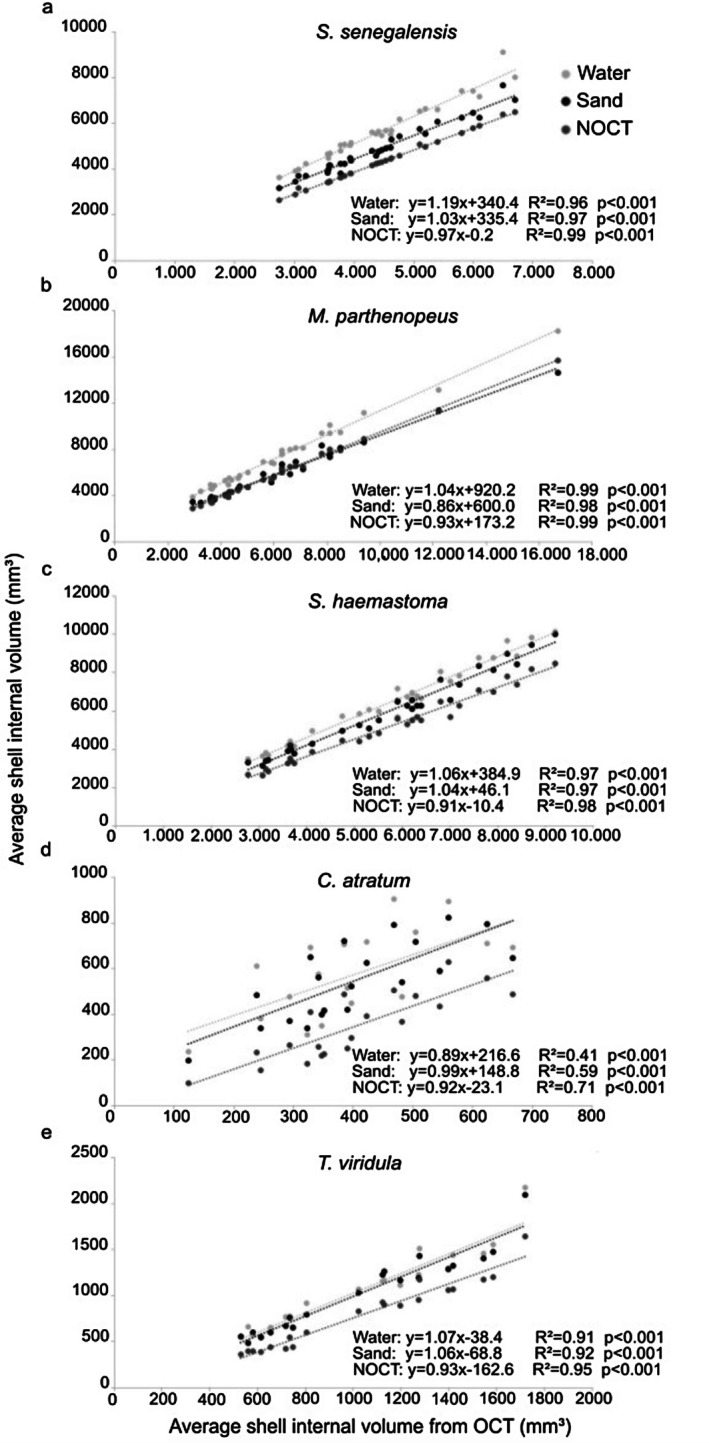
Relationship between mean estimated volumes (mm^3^) by optimized CT (OCT) method and mean estimated volumes (mm^3^) of the three methods (water, sand, and nonoptimized CT (NOCT)) for: (a) *Siratus senegalensis*; (b) *Monoplex parthenopeus*; (c) *Stramonita haemastoma* ; (d) *Cerithium atratum* ; and (e) *Tegula viridula*.

**FIGURE 7:**
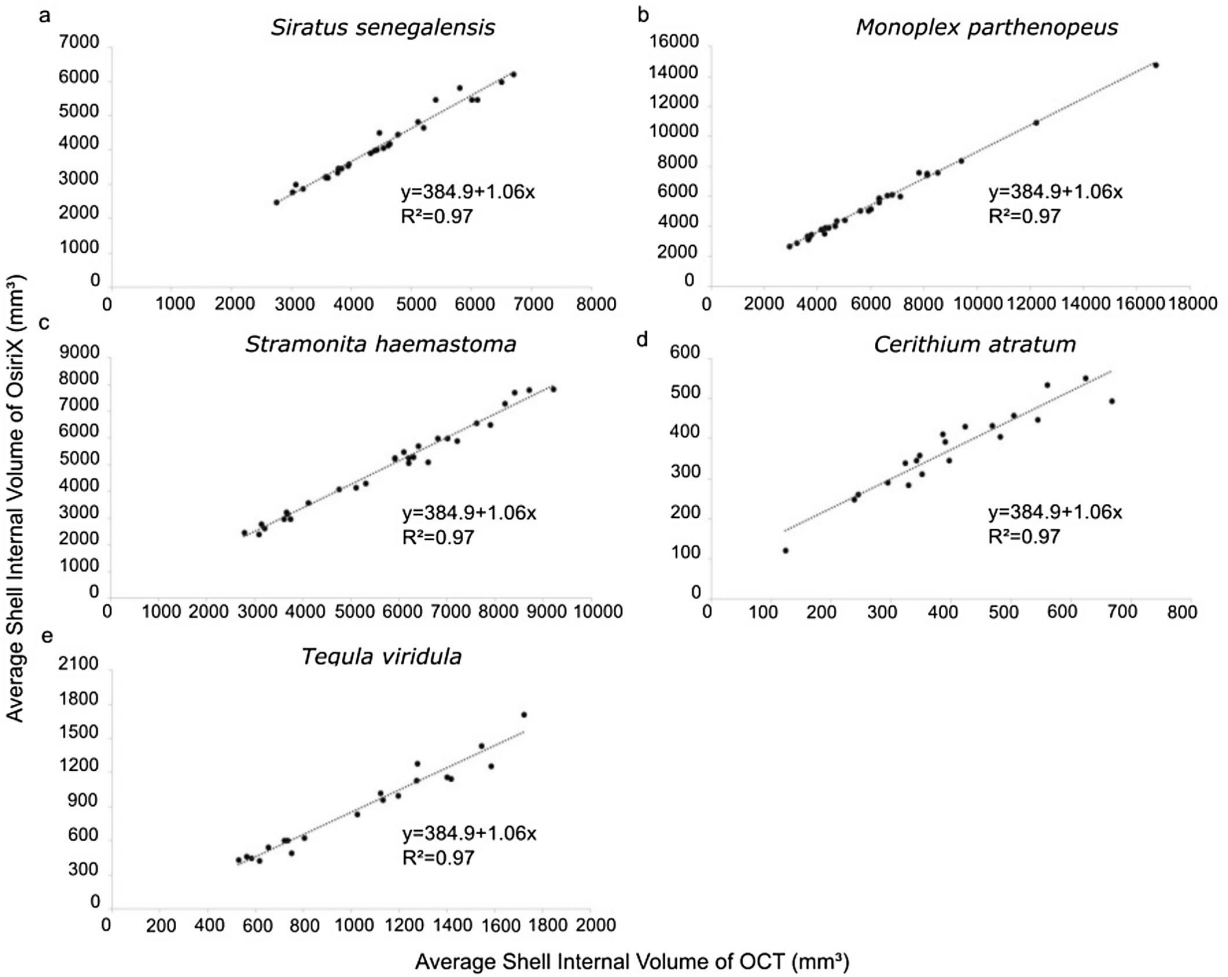
Pearson correlations between mean estimated volumes (mm^3^) estimated from the optimized CT (OCT) method and mean estimated volumes (mm^3^) derived from the software OsiriX MD for: (a) *Siratus senegalensis*; (b) *Monoplex parthenopeus*; (c) *Stramonita haemastoma* ; (d) *Cerithium atratum* ; and (e) *Tegula viridula*.

All methods yielded a high coefficient of determination (*r* ^2^ = 0.97). For the three large shell species with medium spires (Figures 6b,c and 7a), the variability in estimated volumes obtained through OCT and OsiriX MD was lower than that of shells with size variation (Figures 6e and 7d). Among the larger shell species, *S. haemastoma* showed higher variability (Figure 7c), while among shells with size variation, *C. atratum* showed higher variability than *T. viridula* (Figures 6e and 7d). These results indicate that the volumes estimated by OCT and the software are equivalent.

Reproducibility across the different software (expressed by the ICC values) was high for all species (*r* > 0.99): *S. senegalensis* (*F* = 1418.36, *r* = 0.9986, *p* < 0.001); *M. parthenopeus* (*F* = 688.07, *r* = 0.9971, *p* < 0.001); *S. haemastoma* (*F* = 883.98, *r* = 0.9977, *p* < 0.001); *C. atratum* (*F* = 268.43, *r* = 0.9926, *p* < 0.001); and *T. viridula* (*F* = 980.16, *r* = 0.9986, *p* < 0.001). These results indicate that the different volume measurements were equivalent within each shell and moreover emphasize the applicability of the OsiriX MD. Indeed, the estimates derived from the software were comparable to OCT methods for large species *S. senegalensis* and *M. parthenopeus* (i.e., OCT = OsiriX), but 12% lower than OCT for *S. haemastoma* . Focusing on shell species of contrasting architecture (low and high‐spired) with size variation, OsiriX estimates were comparable to OCT only for *C. atratum* shells but yielded lower average volume than OCT for *T. viridula* (23%).

**FIGURE 8:**
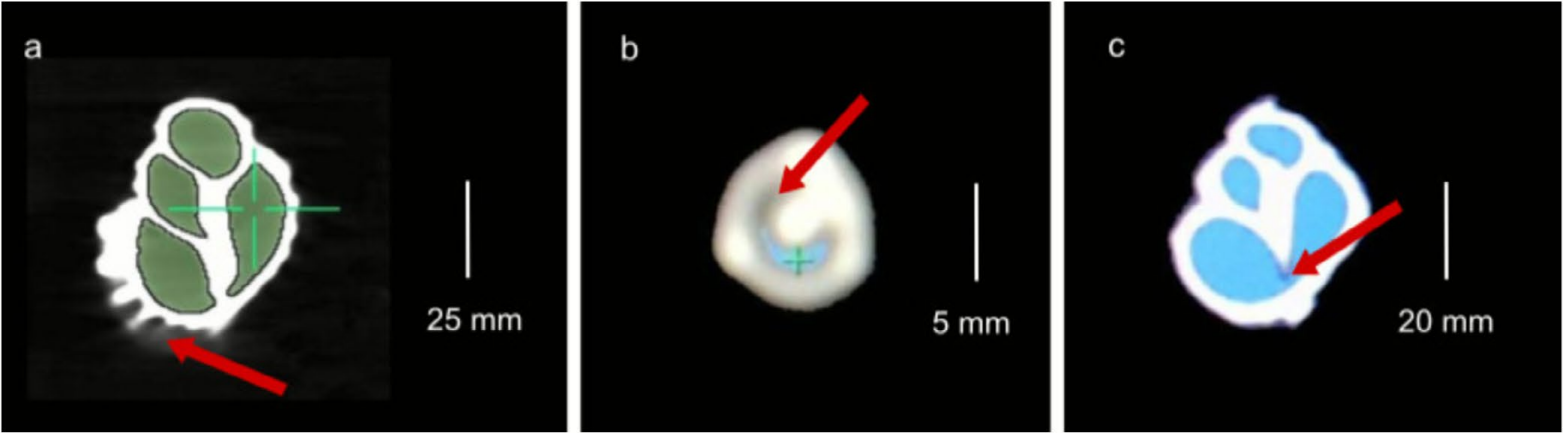
Artifacts in CT images detected in the analyzed images (red arrows). (a) Beam hardening artifact with a false bright in the periphery of sagittal slice from *Monoplex parthenopeus* shell (image from OsiriX MD); (b) Partial volume artifact with a blurring between the shell wall (the “Edge Effect”) in axial slice from *Cerithium atratum* shell (image from CT Viewer software); and (c) Partial volume artifact causing the edge effect in sagittal slice from a *Siratus senegalensis* shell (image from CT Viewer software).

In addition, we recorded two different artifacts during image manipulation for both CT Viewer and OsiriX (Figure 8). One involved a bright light bordering the external shell structure (the “beam hardening” artifact; Figure 8a), but this did not affect the volume measurements. The second was characterized by a blurring near or between structures of different density (the “partial volume averaging” artifact), which induces an overestimation of size for some structures and reduces volume estimation (Figures 7c and 8b). This artifact was evidenced in both software packages, although it was most frequently observed during image manipulation using OsiriX for the species *T. viridula* , *C. atratum* (Figure 8b), and *S. senegalensis* (Figure 8c).

## 4 Discussion

Hermit crabs depend on empty gastropod shells to protect their noncalcified abdomen (Hazlett 1981; Turra et al. 2005), and the internal volume of the gastropod shells has implications for the hermit crabs that inhabit them (Ragagnin et al. 2018). The increasing availability and flexibility of imaging techniques present opportunities for targeted analysis with respect to biological studies (Gutiérrez et al. 2018), especially when these produce more comparable, precise, and/or accurate results (Gutiérrez et al. 2018; Ragagnin et al. 2018), including volume estimates. It is possible because CT allows access into internal structures of the organisms (Kalender 2006, 2011), and because it is an automated technique, which makes it less susceptible to human error (Gutiérrez et al. 2018).

Previous studies have shown that shell species, size, and the method employed (water, sand, or CT) can influence volume estimates and interpretations of internal shell structure (Ragagnin et al. 2018). Our findings corroborate these sources of variability, particularly when comparing approaches such as OCT and OsiriX. In general, our findings showed that an OCT method presented lower estimates than water for the majority of shell species (except for *T. viridula* ), but equivalent estimates to sand for all species tested (except for *S. senegalensis* and *C. atratum* ). The volume estimates obtained from OCT were 10%–30% lower than the water method and 8%–27% lower than the sand method. While the volumes obtained from NOCT were 24%–20% lower than the water method and 12%–37% lower than the sand method. Although both CT methods provided lower volume estimates than the methods traditionally used in ecological studies (Fotheringham 1976; Bertness 1981; Hazlett et al. 2005), the advantages afforded by CT include the possibility of standardizing measurements which improve overall precision.

The estimates obtained through OCT are closer to those obtained from the water method; therefore, we suggest the application of OCT for similar studies and organisms, such as fishes, stromatolites, coral structures, and even some plant structures, such as xylem or sclerenchyma. Furthermore, we hypothesize that the higher volumes obtained using the water method could be due to pores in the shell wall that may absorb some of the water that filled the shell cavity, creating a type of “sponge effect.” This phenomenon could explain the higher estimates obtained using water, but which do not appear to occur using the CT approach.

The manipulation of images showed that shell surface ornaments affected the gray scale of the CT images (DeMAIO 2011), which may explain why estimates derived from the OCT and sand methods are similar for species with less ornamentation, as for *S. haemastoma* and for shells with small elevations, such as *M. parthenopeus*. For species with princles, such as *S. senegalensis* , OCT did not differ from the NOCT, suggesting that for these types of species the two CT methods generate similar results. In addition, images from *S. senegalensis* specimens presented more artifacts, which suggests that princles may also influence the precision of volume estimates.

Compared to the NOCT, the OCT provided higher volume estimates for less ornamented species, *S. haemastoma* and *T. viridula* . Also, the OCT yielded equivalent estimates as NOCT for the other middle‐spired shells, *M. parthenopeus, S. senegalensis*, and for the high‐spired shell, *C. atratum* . This could be due to artifacts emerging in the respective CT images, which suggest that OCT is not preferable for highly ornamented species (as in Ragagnin et al. 2018) but could be a new and viable approach for less ornamented species, including *S. haemastoma* .

There are four types of artifacts that can emerge from CT images (Popilock et al. 2008; Boas and Fleischmann 2012) which are generated by several factors (Kalender et al. 1987; Barret and Keat 2004; Popilock et al. 2008; Boas and Fleischmann 2012; Esmaeili et al. 2012). Two of these artifacts were observed in the present study, one being “beam hardening” (Popilock et al. 2008) and the other being “partial volume averaging” (Kalisz et al. 2016). Beam hardening was identified by a false bright light around the periphery of a structure (Popilock et al. 2008). The beam hardening artifact, however, did not influence volume measurements because it occurs on the external region of the shell and was evident in images from both CT Viewer and OsiriX MD software. Partial volume averaging, however, appears as a blurring near or between structures having markedly different densities. Consequently, some structures, including those comprising calcium and lumens, result in a volume overestimation (Kalisz et al. 2016). Image blurring may overestimate the size of structures comprising high density, such as mollusk shells that are formed by deposits of calcium carbonate (Lowenstam 1981; Dauphin and Denis 2000). In the present study, this artifact likely influenced volume estimates because of a considerable loss of internal air, and consequently underestimating results and leading to the “Edge Effect,” described by Ragagnin et al. (2018). This artifact was more frequently observed for small shells including *T. viridula* and *C. atratum* and for shells with prickles and hips species. Furthermore, these types of shells were more susceptible to artifacts than the other shell architectures analyzed. This artifact was evident for both software platforms (CT Viewer and OsiriX MD) and was most frequently observed in the images manipulated in OsiriX MD. The image acquisition evidenced structures with split ends, which may cause such artifacts (Barret and Keat 2004) and therefore, we hypothesized that when the x‐rays pass through the ornaments, prickles, and hips and then through the shell wall, this influences the latter image acquisition of the shell wall. Our initial hypothesis predicted that adjusting the settings employed during CT (i.e., OCT) could influence the quality of images and improve volume estimates (higher values with reduced variability) because it was also observed that the manipulation of the greyscale influences the partial volume averaging.

For species with size variation, the effect of architecture could be explained by the pattern of the spires and the specimen size. For example, larger shells of *C. atratum* were easier to measure than smaller shells because they had fewer artifacts and consequently less “Edge Effect,” making the distinction between shell wall and air more apparent. The OCT methods provided high volume estimates for *T. viridula* , which were equivalent to those obtained using the water and sand methods and indicated comparable outcomes between traditional and novel CT methods. This outcome could be explained by the less‐spired architecture of *T. viridula* (presumably exhibiting fewer artifacts), while for *C. atratum* both OCT and NOCT gave equivalent estimates as OsiriX MD.

The “Edge Effect” was most observed in *Cerithium atratum* and *T. viridula* , especially in the smaller and high‐spired shells such as *C. atratum* , which has been previously recorded (Ragagnin et al. 2018). Thus, it is evident that the overall size and spire pattern of this species may produce more artifacts. For this reason, the volume measurements obtained using OCT are equivalent to those derived using NOCT. Nonetheless, for the less‐spired species *T. viridula* , only size was important for the emergence of artifacts, since the volume estimates obtained using OCT were equivalent to those derived from the sand method. Therefore, for this species, the artifacts did not influence the volume measurements.

The influence of size on volume estimates was expected because the smaller the shell, the more difficult it is to measure its volume. CT images showed more partial volume averaging artifacts in smaller shells, which increased the “Edge Effect.” But the variability in estimated shell volumes was higher for large *C. atratum* than for smaller shells for all methods, including by the OsiriX software. On the other hand, there was lower variability across all size classes and methods for *T. viridula* . Considering the OCT method, these results indicate a greater influence of size and architecture on volume estimates for *C. atratum* because of more frequent artifacts observed for this species than for *T. viridula* .

The OCT protocol based on model shells did not allow for its direct transposition to other shells of the same type or size. This finding indicates that the method is not as practical or rapid as initially expected, since optimization would need to be performed individually for each shell. Therefore, although OCT can yield accurate results, its application requires careful adjustment, limiting its efficiency when dealing with a large number of specimens or morphologically diverse shells. Because of that, for species with principles, hips, or high‐spired shells, we recommend the use of NOCT, which is less time consuming.

The partial volume averaging artifact was observed more frequently in the OsiriX MD software, leading to a pronounced “Edge Effect” in the manipulated images. This could be one driver of the variation in results found between this proprietary software and OCT for *T. viridula* . However, the volume estimates obtained using OsiriX did not differ from those obtained using both CT methods for the middle‐spired large shells (*M. parthenopeus* and *S. senegalensis* ) and for the high‐spired species *C. atratum* . Moreover, the high ICC (Intraclass Correlation Coefficient) values derived for volume estimates in OsiriX indicate the high reproducibility of the quantitative analyses, supporting the conclusions of several studies that have employed this software (Yamauchi et al. 2010; Kim et al. 2012; Shyu et al. 2015). In addition, for *M. parthenopeus*, *S. senegalensis* , and *C. atratum* the volumes were similar to those derived from OCT. Overall, this suggests that the OsiriX output is comparable to that obtained from the standard proprietary software, which is widely known to have high reliability and accuracy (Yamauchi et al. 2010; Kim et al. 2012; Molinari et al. 2014; Shyu et al. 2015; Gumsheimer et al. 2017). Because of that, we therefore advocate that the proprietary software should not be the only option used for shell volume estimation, and an option could be OsiriX, which produces comparable and reliable results. Future studies may explore and validate additional software alternatives, such as 3D Slicer, broadening the accessibility and reproducibility of the CT method.

Increasing the display WW also reduces the partial volume averaging artifact (Kalisz et al. 2016). The OCT employed the most appropriate WW for each shell specimen, which was used in subsequent estimates of volume. Thus, an optimized setting for the tomography parameters WW and WL was hypothesized to produce more accurate calculations of the shell internal volume and avoid artifacts such as “Edge Effects.” This artifact occurs because the parameters are related to the contrast between two adjacent pixels and to the density of an object. Therefore, our results show that different parameter combinations provide different internal volume estimates, which relate to the error associated with the CT method. Decreasing the thickness of the slices, decreasing the collimated detector width, or increasing the voltage tube are viable solutions to minimize the partial volume averaging (Kalisz et al. 2016). Another solution can be decreasing the voxel size (Simões and Campos 2013), which can have a similar outcome. One cause of this artifact can be the representation within the same voxel of structures having different densities (Kalisz et al. 2016). Using a lung filter during analyses of the CT images may improve the distinction between dense structures and air and have the advantage that it can be applied to previously acquired CT images.

Overall, the presence of CT artifacts and limitations to our ability to optimize available CT methods means that new approaches to image analysis should be tested and applied to studies investigating the volume of structurally complex organisms, but also to other ecological issues related to the structure and function of the natural world. One new approach that could be applied to such studies could be microtomography (micro‐CT), which has already been proven to be useful across a range of biological (Alba‐Tercedor et al. 2016; Shahmoradi and Swain 2016; Solomon et al. 2016), anthropological (Reinholt et al. 2009; Abel et al. 2011), and geological studies (Taylor et al. 2015; Sahoo et al. 2016). This method uses thinner slices and employs higher resolution imagery than CT methods, which could reduce the influence of artifacts. Consequently, this method may be a more useful and precise means of deriving reliable measurements of shell internal volume.

## 5 Conclusion

In conclusion, different parameter combinations of WW and WL result in different estimates of internal volume and therefore CT instrument settings should be carefully adjusted to avoid inaccurate measurements, which can be time‐costing and could make it unviable to use the OCT. The shell size and ornamentation are the main factors influencing volume estimation, indicating that while a general CT protocol can be applied across gastropods, its use should be adjusted according to shell morphology rather than species identity. For highly ornamented or high‐spired shell species, we recommend the use of NOCT methods over OCT, since volume estimates were generally comparable and thus the additional effort appears unjustified. However, for less ornamented or lower‐spired shell species, the application of an OCT approach could improve estimates and thus warrant consideration. In addition, we conclude that currently available proprietary software OsiriX MD could be a viable alternative to the standard proprietary software to investigate internal structures and derive volume estimates.

The partial volume averaging artifact observed for some CT images in the present study was likely the main cause of the “Edge Effect” described by Ragagnin et al. (2018) and could lead to an underestimate of volume measures. We suggest that future studies could take advantage of additional tools such as filter lungs applied to CT images that could be tested for their capacity to minimize artifacts. We also suggest detailed experimental trials of new methods such as computed microtomography (micro‐CT) to improve this suite of approaches and provide further understanding of the results of the present study. Since micro‐CT is likely to provide thinner slices and higher resolution imagery than regular CT scans, it could reduce any artifacts and provide greater confidence in the data generated to answer biological questions. Thus, this method reveals the potential for more precise data and should be considered in the future.

## Author Contributions

**Andreza Caroline Caiero:** conceptualization (lead), data curation (lead), investigation (lead), methodology (lead), project administration (lead), resources (lead), software (lead), writing – original draft (lead), writing – review and editing (lead). **Marilia Nagata Ragagnin:** conceptualization (equal), formal analysis (supporting), investigation (supporting), methodology (supporting), software (supporting), supervision (supporting), validation (supporting), visualization (supporting), writing – original draft (supporting), writing – review and editing (supporting). **Cláudio Campi de Castro:** data curation (equal), methodology (supporting), software (supporting), validation (equal). **Marcelo Gusmão Paraíso Cavalcanti:** formal analysis (supporting), software (supporting), validation (equal), writing – original draft (equal), writing – review and editing (equal). **Daniel Gorman:** visualization (supporting), writing – original draft (supporting), writing – review and editing (supporting). **Alexander Turra:** conceptualization (supporting), formal analysis (lead), funding acquisition (lead), investigation (supporting), methodology (supporting), project administration (supporting), resources (supporting), supervision (supporting), validation (supporting), writing – original draft (supporting), writing – review and editing (supporting).

## Funding

This work was supported by grant #2016/22759‐7, Sao Paulo Research Foundation (FAPESP).

## Conflicts of Interest

All the authors declare that they have no financial or nonfinancial interests that are directly or indirectly related to the submission or publication of this work. The authors also declare that they have no professional and personal interests or personal beliefs related to this work, as well as having no personal relationships with the editorial board of this journal. This research was funded by the São Paulo Research Foundation (FAPESP) under grant number #2016/22759–7, São Paulo Research Foundation.

## Supporting information


**Data S1:** ece372650‐sup‐0001‐DataS1.xlsx.

## Data Availability

All the required data are uploaded as Data S1.
